# Anti-carbamylated protein autoantibodies associated with mortality in Spanish rheumatoid arthritis patients

**DOI:** 10.1371/journal.pone.0180144

**Published:** 2017-07-03

**Authors:** Laura Vidal-Bralo, Eva Perez-Pampin, Cristina Regueiro, Ariana Montes, Rosana Varela, Maria Dolores Boveda, Juan J. Gomez-Reino, Antonio Gonzalez

**Affiliations:** 1 Laboratorio Investigacion 10 and Rheumatology Unit, Instituto de Investigacion Sanitaria-Hospital Clinico Universitario de Santiago, Santiago de Compostela, Spain; 2 Unit of Diagnosis and Treatment of Congenital Metabolic Diseases, Department of Pediatrics, Instituto de Investigacion Sanitaria-Hospital Clinico Universitario de Santiago, Santiago de Compostela, Spain; 3 Department of Medicine, University of Santiago de Compostela, Santiago de Compostela, Spain; Keio University, JAPAN

## Abstract

Patients with rheumatoid arthritis (RA) have an increased mortality rate that is associated with the presence of RA-specific autoantibodies in many studies. However, the relative role of rheumatoid factor (RF), anti-CCP antibodies and the most recently established RA-autoantibodies, directed against carbamylated proteins (anti-CarP antibodies), is unclear. Here, we have assessed the role of these three antibodies in 331 patients with established RA recruited from 2001 to 2009 and followed until November 2015. During this time, 124 patients died (37.5%). This death rate corresponds to a mortality rate 1.53 (95% CI 1.26 to 1.80) folds the observed in the reference population. We used for analysis of all-cause mortality the Cox proportional hazard regression model with adjustment for age, sex and smoking. It showed a trend for association with increased mortality of each of the three RA autoantibodies in antibody-specific analysis (hazards ratio (HR) from 1.37 to 1.79), but only the HR of the anti-CarP antibodies was significant (HR = 1.79, 95% CI 1.23 to 2.61, p = 0.002). In addition, the multivariate analysis that included all autoantibodies showed a marked decrease in the HR of RF and of anti-CCP antibodies, whereas the HR of anti-CarP remained significant. This increase was specific of respiratory system causes of death (HR = 3.19, 95% CI 1.52 to 6.69, p = 0.002). Therefore, our results suggest a specific relation of anti-CarP antibodies with the increased mortality in RA, and drive attention to their possible connection with respiratory diseases.

## Introduction

Rheumatoid arthritis (RA) is an autoimmune systemic disease that affects primarily diarthrosis (joints with a wide range of movement and covered with synovial tissue) with inflammation, pain, disability and deformity [1,2]. It can also include extra-articular manifestations involving the lungs, the blood vessels or the skin. Other characteristics of the disease are systemic inflammation and alterations of the immune system, including the production of specific autoantibodies. RA is also associated with a notable increase in the death rate that has been quantified at about double the standard mortality rate of the population [3–6]. This increase has dire consequences as it shortens the life expectancy of patients with RA by about 10 years. The excess mortality is due to multiple comorbidities, which include cardiovascular and cerebrovascular diseases, infections, lymphoma, and gastrointestinal diseases, as well as, a group of less common causes of death [3,4,6,7]. The mechanisms leading to increased mortality are not completely understood, although inflammation is associated with cardiovascular disease, and the cumulative burden of disability, decrepitude, pain and treatment side effects is suspected to cooperate with other comorbidities [3,4,7,8]. Accordingly, mortality is associated with RA disease activity and severity. These associations could, in turn, account for the increased mortality observed in patients with RA-specific autoantibodies [3,4,6,7,9–13], because they show a more severe disease [14–17]. However, a more direct effect of the anti-CCP antibodies could be also involved [18].

The first identified RA-specific autoantibody is rheumatoid factor (RF) [1,2]. It consists of IgM antibodies directed against the Fc portion of IgG. It is present in about 70% of the RA patients, but also in a small fraction of patients with other inflammatory diseases or healthy subjects. More recently, several antibodies directed against post-translational modifications of proteins have been identified as RA-specific. The first antibodies of this type that were characterized recognize citrullinated proteins. They are analyzed as anti-cyclic citrullinated peptide antibodies (anti-CCP). These antibodies are very specific of RA, participate in its pathogenesis and are useful diagnostic, prognostic and treatment biomarkers [1,2]. Subsequently, the anti-carbamylated protein antibodies (anti-CarP) were discovered [19–23]. They are rarely assessed because we do not have yet a standardized assay for these antibodies. However, there is already evidence of their involvement in RA pathogenesis and of their possible utility as biomarkers.

As mentioned, the presence of RA autoantibodies has been associated with mortality in multiple studies [3,4,6,7,9–13]. However, most studies have included only one antibody or have not compared their relative roles. In the minority of reports comparing antibodies, the results are often discordant [9–13]. This heterogeneity of results is evident for RF and anti-CCP antibodies, which are the most frequently analyzed. The relative association with mortality has been reported as a significant association that is exclusive or dominated by the presence of RF [9,12,13], or the reverse, a significant association dominated by the presence of anti-CCP antibodies [11], or in between, with the two antibodies contributing additively to a significant association [10]. A similar situation was observed in the single study addressing the anti-CarP antibodies, where the relative weight of the antibodies was different in each of the three patient cohorts, one showed an association with mortality dominated by RF, a second cohort an association dominated by anti-CarP antibodies, and in the third the association with mortality involved the additive contribution of the three antibodies [9].

With these antecedents and considering the need of more information, we have addressed the relative association of the three RA autoantibodies with mortality in our RA patients. In particular, we wanted to address the anti-CarP antibodies, as the less studied of the three antibodies. In addition, we were specifically interested in testing if the associations with the different autoantibodies were statistically different, as opposed to simply of different strength, and if they showed an additive effect irrespective of the autoantibody type. After completing the analysis, we have found that only the anti-CarP antibodies were significantly associated with mortality, but this association was not statistically different from the trends observed with the other two autoantibodies. In addition, the anti-CarP antibodies association with mortality was dominant over the association with the number of antibodies, and showed specificity for deaths attributed to respiratory diseases.

## Materials and methods

We have included patients with established RA meeting the 1987 American College of Rheumatology classification criteria in this study [24]. Their recruitment took place from 2001 to 2009 at the Rheumatology Unit in our Hospital. We also obtained samples, demographic data and smoking information, together with the written informed consent. The Ethics Committee for Clinical Research of Galicia approved the study. All patients were Spanish Caucasians. Information on survival since recruitment until November 2015 was collected from the electronic medical records of the patients, which are kept across all levels, from primary care to tertiary level hospitals, of the public and private health centers of Galicia in the IANUS system [25]. The last entry for each patient was the date of death or of the last follow-up visit. Causes of death were obtained from the same IANUS system in 68 patients and from the Galician Death Registry in the remaining 56 patients according with the International Classification of Diseases (ICD) 9 and 10 revisions. They were grouped in four categories: circulatory system, respiratory system, neoplasms and other, which included the remaining causes of death of less frequency.

Autoantibodies were determined in serum samples taken at the time of recruitment. IgM RF was measured by rate nephelometry (IMMAGE Immunochemistry System; Beckman Coulter). Anti-CCP antibodies were assayed by ELISA (Euro-Diagnostica, Malmö, Sweden) applying a cutoff level of 5 units/ml. Anti-CarP antibodies were assessed by homemade ELISA with *in vitro* carbamylated fetal bovine serum following the protocol described by Shi *et al*. [19], as we have previously reported in detail [23]. The cutoff level was determined using 208 healthy controls at 138.61 arbitrary units against serial dilutions of a pool of positive sera.

We calculated standardized mortality ratios (SMR) of the patients with RA relative to the sex- and age-specific mortality rates observed in the province of A Corunna, where most of the patients in this cohort have their residence. The year of reference was 2008, the mid-year of follow-up. Mortality rates of the population were obtained from the Spanish National Institute of Statistics that provides detailed actuarial tables [26]. For the association analysis, we used Cox proportional hazard regression models for all-cause mortality or for specific causes of death considering age, sex and smoking status as covariates. Hazard ratios (HR) of RF, anti-CCP and anti-CarP positivity were obtained in antibody-specific and, antibody-combination models. In addition, number of autoantibodies and the logarithm-transformed antibody titers were considered in separate analyses. For graphical representation, we obtained survival curves with the Kaplan-Meier product limit analysis, which is a univariate analysis (not adjusted for any confounding variable). In addition, proportional Venn diagrams were drawn with eulerAP [27]. Finally, concordance in status between antibodies was assessed with the Goodman and Kruskal gamma (γ). All analyses were done with Statistica 7.0 (Stat Soft, Inc).

## Results

**Fig 1:**
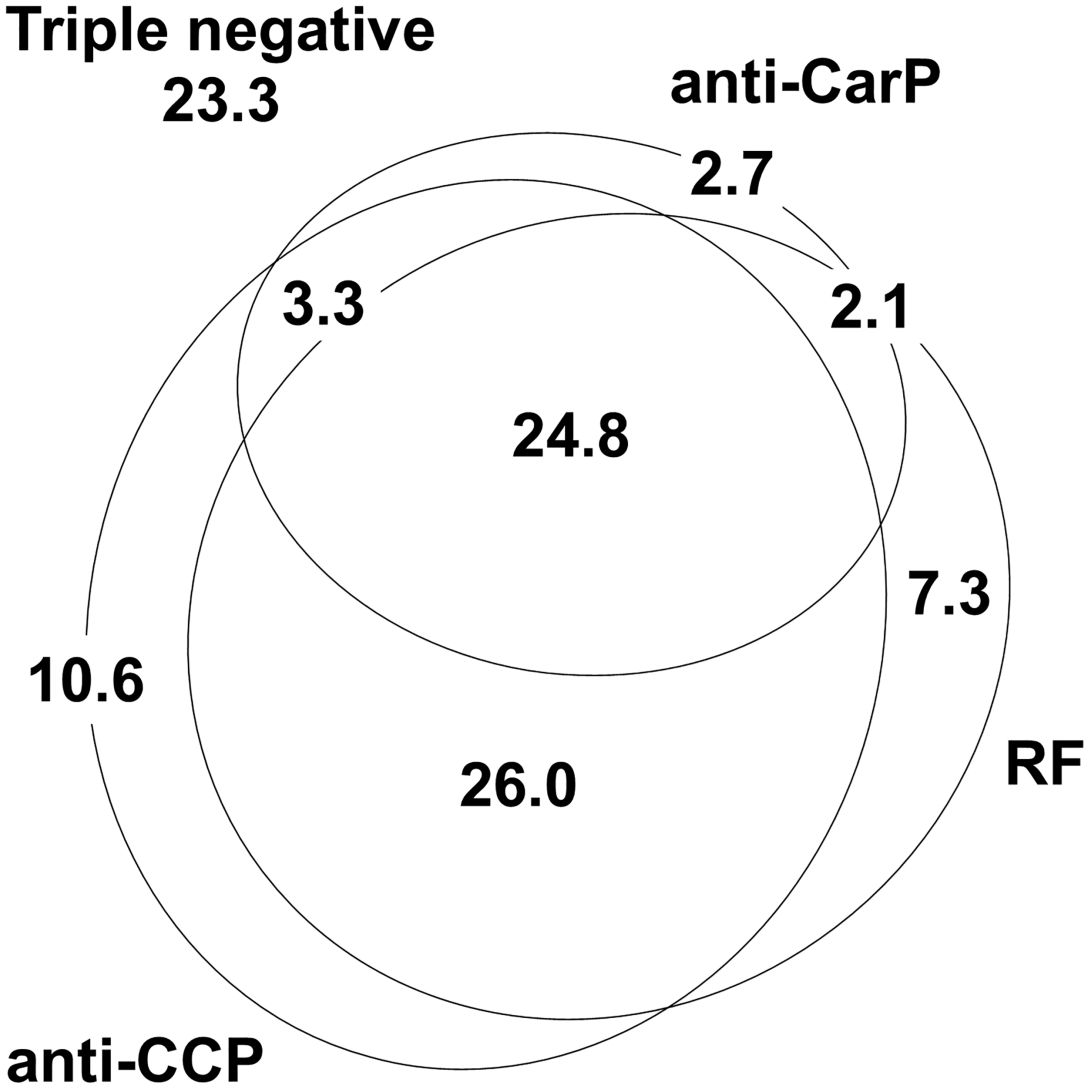
Distribution of patients with RA positive for the different autoantibodies. A proportional Venn diagram representing percentages (%) of patients in each of the antibody strata: anti-CCP positive in the lower left oval, RF positive in the lower right oval and anti-CarP positive in the upper one. Percentage of triple negatives is shown outside the Venn diagram in the upper left corner.

The 331 patients included in this study were recruited from the outpatient Rheumatology clinic without any selection except fulfilling the ACR classification criteria. They showed characteristics of established RA as shown in Table 1, with an excess of women, advanced age and long time since the start of the first disease symptoms (Data in S1 Table). They were also typical in the fraction showing RA-specific autoantibodies: RF = 60.1%, anti-CCP = 64.7% and anti-CarP = 32.9%, and in the overlap between them (Fig 1), corresponding with concordances of γ = 0.82 between anti-CCP and RF, 0.66 between anti-CCP and anti-CarP, and 0.64 between RF and anti-CarP (p for all of them < 2.0 x 10^−16^). The frequency of ever smokers was low (Table 1), but characteristic of the smoking habit in the area covered by our hospital. Smoking showed a notable difference between women and men (5.3% of the women, 95% CI 3.1 to 8.9%, *vs*. 52.3% of the men, 95% CI 41.9 to 62.6%).

**Table 1:** Main characteristics of the patients included in this study.

Feature	Value
Number	331
Women, % (95% CI)	74.0 (69.0–78.5)
Age of disease onset, mean (SD)	52.4 (13.3)
Age at recruitment, mean (SD)	68.7 (8.1)
Erosions, % (95% CI)	67.2 (61.9–72.1)
Ever smoker, % (SD)	17.5 (13.8–22.0)
Rheumatoid factor, % (95% CI)	60.1 (54.8–65.3)
Anti-CCP, % (95% CI)	64.7 (59.4–69.6)
Anti-CarP, % (95% CI)	32.9 (28.1–38.2)
Time of follow-up, mean (SD)	9.0 (3.5)
Deaths, % (95% CI)	37.5 (32.4–42.8)

Recruitment was done from 2001 to 2009 and survival data were collected until November 2015. This corresponds to a mean follow-up of 9.0 years (SD = 3.5 years) and to 2991 person-years of survival data. In this period, 124 patients have died (Table 1), which correspond to a crude mortality rate of 41.5 ‰ (95% CI 34.9 to 49.3 ‰). This number of deaths reflects an increased mortality as shown by the SMR of 1.53 (95% CI 1.26 to 1.80), which is the excess observed mortality among the patients relative to the observed in the reference population. The main causes of death (individual level supporting information provided in S1 Table) were diseases of the circulatory and of the respiratory system (36 deaths in each), followed by neoplasms (18 deaths) and infections (13 deaths).

**Fig 2:**
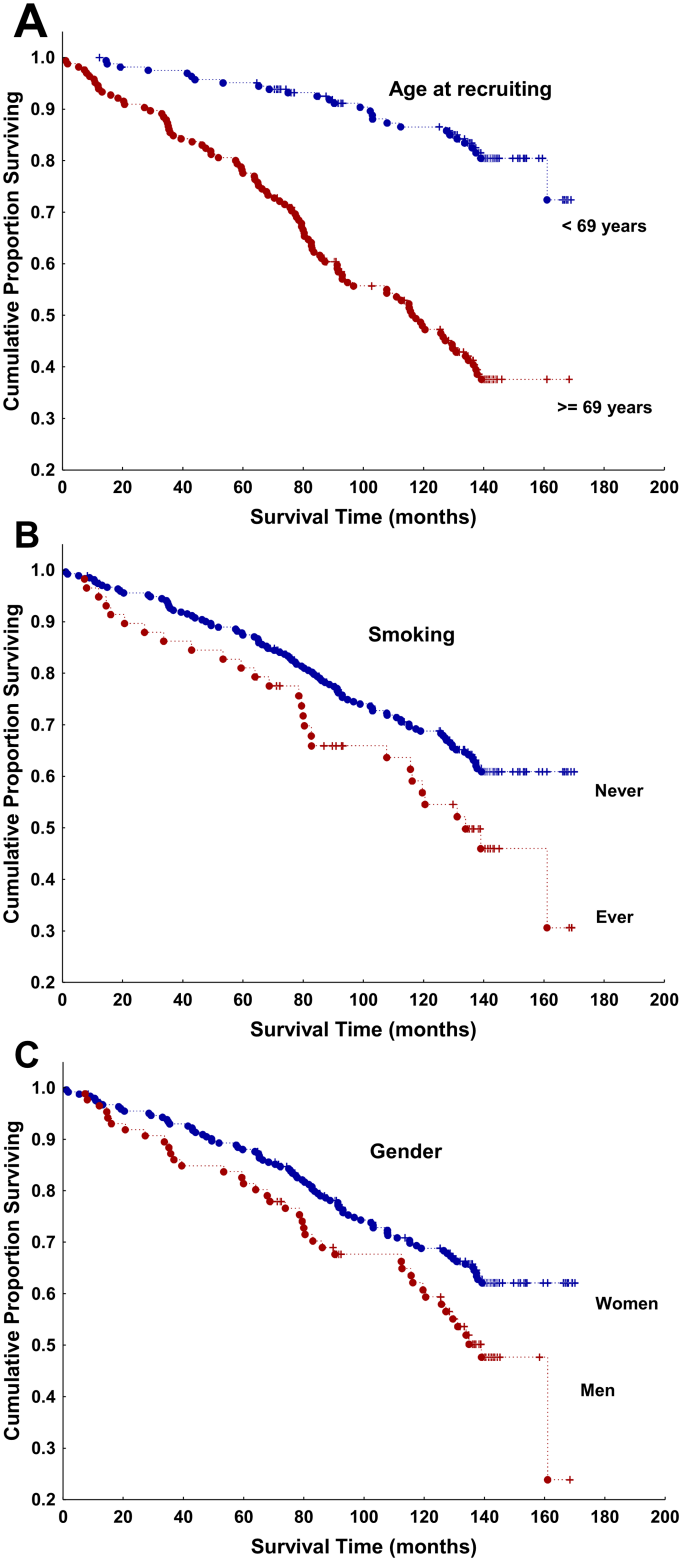
Kaplan-Meier survival curves of all patients with RA stratified by demographic factors. (A) Stratified by age in relation with the median age at the time of recruitment (red ≥ 69 years, blue < 69 years); (B) stratified by smoking habit (red = ever smoker, blue = never smoker), and (C) stratified by gender (red = men, blue = women). Dots = deaths, crosses = censored data.

Known risk factors were associated with increased mortality in the univariate analyses (Fig 2), old age (HR = 1.13 expressed in years, 95% CI 1.11 to 1.16, p = 2.5 x 10^−24^), smoking (HR = 1.59, 95% CI 1.04 to 2.42, p = 0.03) and male sex (HR = 1.56, 95% CI 1.07 to 2.27, p = 0.02). However, only old age and smoking remained significant in multivariate analysis (old age HR = 1.15, 95% CI 1.12 to 1.18, p = 8.6 x 10^−25^; smoking HR = 2.66, 95% CI 1.58 to 4.48, p = 0.0002; and male sex HR 0.88, 95% CI 0.56 to 1.37, P = 0.6). In contrast, bone erosions (HR = 0.81, 95% CI 0.58 to 1.22, p = 0.4), year of recruitment (HR = 0.98, 95% CI 0.87 to 1.09, p = 0.7) and duration of RA since symptom onset accounting for differences in age (HR = 1.01, 95% CI 0.99 to 1.02, p = 0.2) were not associated with increased deaths. Most patients have already experienced RA before the arrival of the biologic drugs of increased efficacy, around the year 2000, and no differences in HR were observed between those starting before and after this year (HR = 0.94, 95% CI 0.52 to 1.71, p = 0.9).

**Fig 3:**
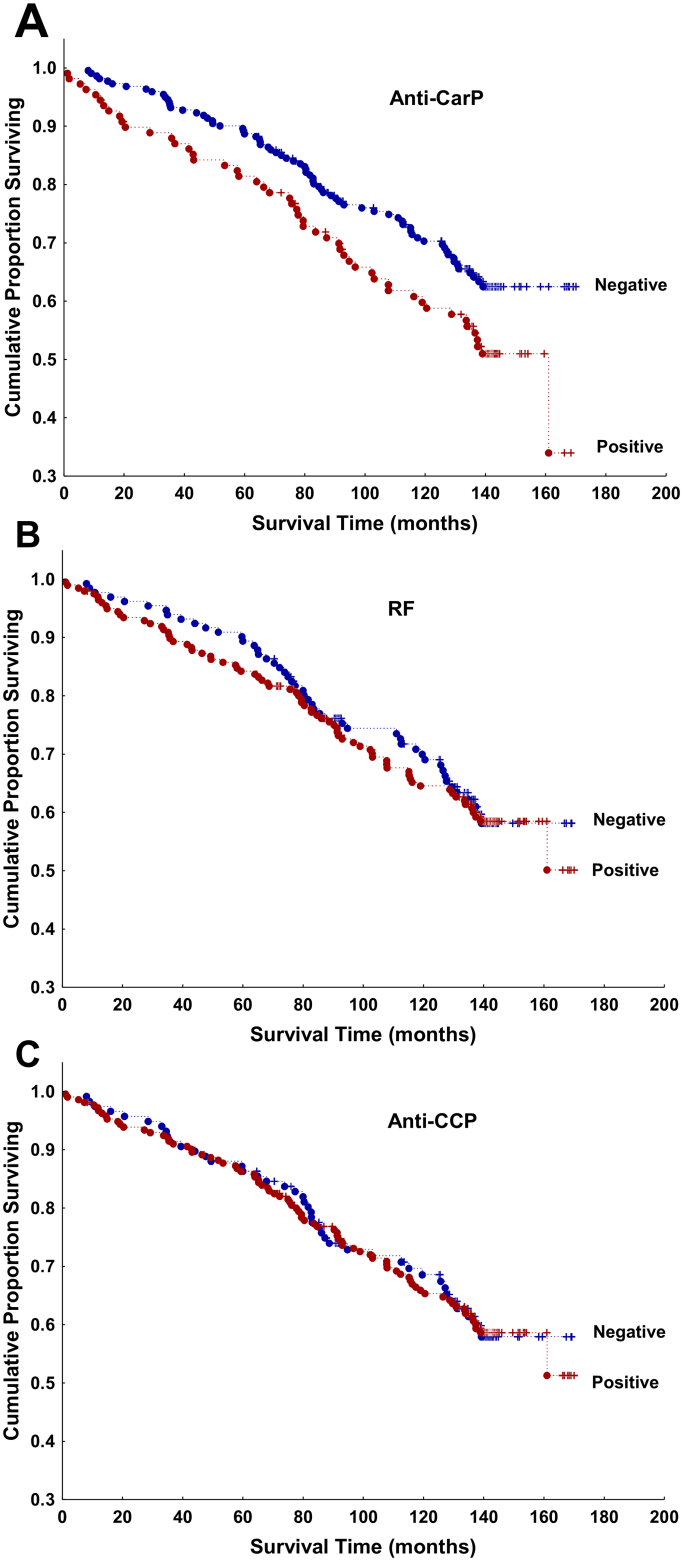
Kaplan-Meier survival curves of all patients with RA stratified by the status of the RA specific antibodies. (A) anti-CarP antibodies, (B) RF, and (C) anti-CCP antibodies. Red = antibody positive, blue = antibody negative, dots = deaths, crosses = censored data. Note that these are unadjusted analyses.

In the antibody-specific analyses, only the anti-CarP antibodies showed a significant association with decreased survival (Fig 3). This result from the unadjusted analysis was reflected in increased HR for anti-CarP positive patients (HR = 1.79, 95% CI 1.23 to 2.61, p = 0.002), with age, sex and smoking as covariates in the adjusted analysis. RF and anti-CCP positive status showed a trend to higher HR in similarly adjusted analyses, but none of them was statistically significant (HR = 1.37, 95% CI 0.94 to 2.00, p = 0.10; and HR = 1.38, 95% CI 0.93 to 2.04, p = 0.11, respectively).

**Fig 4:**
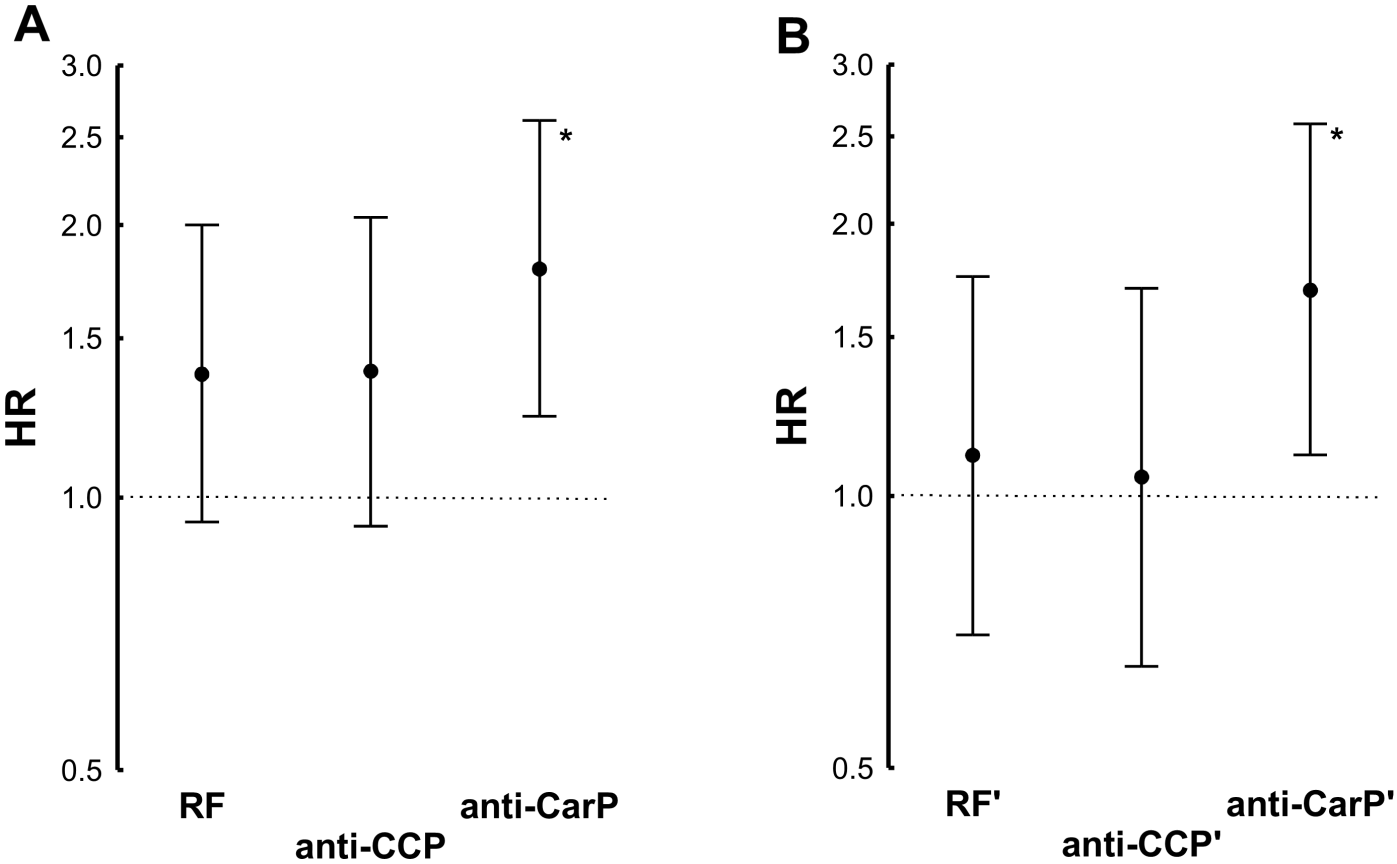
Comparison of the HR obtained by Cox proportional analysis of the three RA autoantibodies. (A) The three antibodies, RF, anti-CCP and anti-CarP, considered each of them separately; and (B) the three antibodies considered conditional on the other two (indicated by the apostrophe, ‘, over them). Error bars correspond to the 95% CI. The asterisks, *, denote significantly increased HR.

The differences between the HR observed with the three antibodies were not significantly different (Fig 4A). However, in a conditional analysis considering the status for the three antibodies (Fig 4B), the trend showed by RF and anti-CCP was notably decreased (HR = 1.11 and 1.05, respectively), whereas the HR of the anti-CarP antibodies remained significantly increased (HR = 1.69, 95% CI 1.11 to 2.58, p = 0.01).

**Fig 5:**
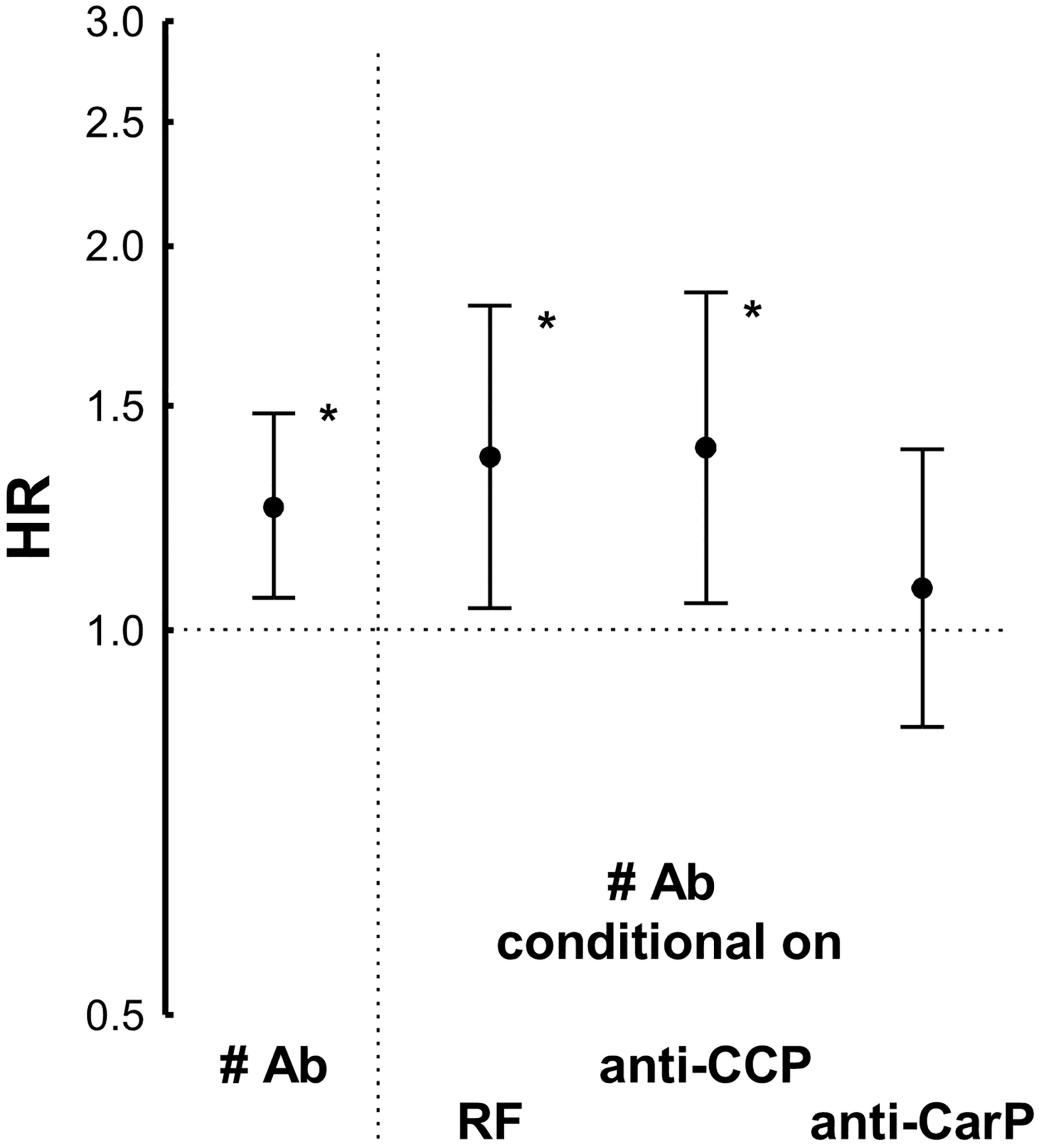
Conditional analysis of the HR observed with the number of autoantibodies. The HR for the number of antibodies (# Ab) obtained in Cox proportional analysis was considered either on itself (to the left of the dotted vertical line) or conditional on the presence of each of the three specific autoantibodies (to the right of the dotted vertical line). The asterisks, *, denote significantly increased HR.

In addition, we assessed the number of positive autoantibodies, either 0, 1, 2 or 3. This variable showed a significant association with mortality (HR = 1.25, 95% CI 1.06 to 1.48, p = 0.008), but it was dependent on the presence of the anti-CarP antibodies, not on the contribution of the other two autoantibodies. These results were obtained by conditional analysis: the number of autoantibodies did not remain associated in analysis conditional on anti-CarP status (HR = 1.08, 95% CI 0.84 to 1.39, p = 0.6), whereas it remained significantly associated, and was nominally more marked, conditional on RF, or on anti-CCP status (Fig 5). Finally, we addressed the autoantibody titers, which were only available for the anti-CCP and anti-CarP antibodies. None of them was associated with a higher HR of death.

When the specific causes of death were considered, only the deaths attributed to diseases of the respiratory system were associated with the anti-CarP antibodies (HR = 3.19, 95% CI 1.52 to 6.69, p = 0.002). This HR was considerably larger than the observed in the all-cause of mortality analysis reported above. The respiratory system group of causes of death included 16 pneumonias and other respiratory infections, 8 interstitial lung diseases that could be attributed to RA, 3 respiratory insufficiencies attributed to COPD, and 9 patients with only disease group level information corresponding to the J00-J99 codes of the ICD10, which we received without detailing. The small number of patients with each disease did not allow to distinguish if any of them was disproportionately contributing to the increased association. Even so, patients with interstitial lung disease showed a nominally increased frequency of anti-CarP antibodies (62.5%). Apart from respiratory diseases, the deaths attributed to neoplasms also showed a trend to increased HR in patients with anti-CarP antibodies (HR = 2.45, 95% CI 0.93 to 6.43, p = 0.07). In contrast, the other two RA autoantibodies did not show association with any of the analyzed causes of death.

## Discussion

Here, we have found that the increased mortality of patients with RA was associated with the presence of anti-CarP antibodies. This association was the only significant among the autoantibodies and clearly dominant over the trends observed with RF and anti-CCP antibodies. In addition, the anti-CarP antibodies were specifically associated with deaths attributed to respiratory diseases.

Only a previous study has analyzed the relation of the three specific RA antibodies with mortality [9]. It showed dominant associations of the anti-CarP antibodies in one of the three cohorts included, but of RF in other and an additive contribution in the remaining cohort. The authors attributed the different outcomes between cohorts to variation in the antibody assays or to heterogeneity in the cohort inclusion settings. These are plausible explanations, although their influence has never been tested directly. The assay method of anti-CarP antibodies is particularly prone to variation between laboratories as not any standardized antigen or ELISA is yet available [19,23]. Also, genetic and environmental factors could have an influence, as already observed in studies of antibodies against specific citrullinated peptides, where large differences in the frequencies of SE alleles and smoking habit between RA sample collections from Sweden, the Netherlands, the UK and Spain, among other countries, have been reported [28–30]. An additional difference between the patients included in Ajeganova *et al*. and our patients is that the first were early RA patients, whereas our patients were of established RA. This difference means that our patients were older and, likely, suffered of more comorbidities. This range of factors could also explain the discrepant results observed in the more numerous studies addressing the relative importance of the anti-CCP and RF association with mortality [9–13]. As mentioned in the introduction, a variety of results has been observed, from the dominant association with RF [9,12,13] to the opposite dominant role of anti-CCP [10,11]. The variability in the relative roles of the RA autoantibodies is not exclusive of mortality, it extends to many other patient characteristics [14,15]. A clear example of recent interest is the controversy over the relative association of anti-CCP antibodies and RF with bone erosions [14,31]. A notable factor in all these conflicting results is the high concordance of status between the antibodies [20,21,23], which likely is a consequence of the epitope spreading that is part of the immune dysregulation leading to RA [22,32,33]. This concordance was observed in our study (Fig 1). It means that only a fraction of the patients, only those showing discordant status, are informative for establishing the relative importance of their association with mortality or any other trait. These considerations invite to a circumspect consideration of the relative importance of the autoantibodies until more evidence becomes available. We think this applies also to our results in spite of the clear dominance of anti-CarP antibodies.

Less abundant are the studies addressing the association of RA autoantibodies with specific causes of death. Only the already mentioned study of Ajeganova *et al*. has addressed this question with the three antibodies considered here [9]. It showed mortality by respiratory diseases and neoplasms associated with RF and mortality by circulatory system diseases associated with anti-CCP antibodies. Other studies have considered the association of causes of death in seropositive patients, but not in a comparative way [6,34–38]. The most common finding has been association with deaths by cardiovascular diseases [34–38], although deaths by respiratory diseases were associated with RF in at least four studies [6,9,34,35]. Therefore, the association of RA autoantibodies with death due to respiratory disease has some support in the bibliography, but the association with the anti-CarP antibodies is unprecedented and should be regarded with caution until replicated. In addition, there are particular limitations that affect any analysis of causes of death and that invite to additional prudence. They include the difficulty to establish the cause of death in many patients, and the grouping of the different causes of death by a mixture of pathogenic mechanisms and organ systems as in the ICD coding [39–41].

The association of increased mortality with the RA autoantibodies could be relevant for two aspects of clinical importance: to help elucidate the mechanisms leading to increased mortality, and to act as a useful biomarker identifying patients at increased risk. Unfortunately, there is not yet clear indication of the antibodies value in any of the two aspects. Regarding the possible mechanisms, there are several hypotheses, but none of them is established [3–8]. As mentioned in the introduction, excess mortality is attributed to a wide array of comorbidities, from cardiovascular to gastrointestinal diseases, and from infections to cancer. This wide array of death causes suggests mechanisms involving decreased overall health, as could result from the burden of inflammation, disability, pain and drug adverse events. Most likely, this is not enough. Together with the overall detrimental effect, there are pieces of evidence identifying mechanisms more directly related to specific causes of death. They include the effect of chronic inflammation in reducing the threshold for atherosclerosis and coronary artery disease [42]; the disorganized immune system as the cause of the increased infections, which could be also exacerbated as a consequence of corticosteroid treatments and other immune modulator drugs [43,44]; and the chronic stimulation of lymphocytes and, again, the dysregulated immune system behind the increased frequency of lymphoma and other cancers. The presence of autoantibodies has often been considered in generic terms, as a feature correlating with a more aggressive or severe disease. This participation is applicable to any of the three RA antibodies because the three correlate with severe RA [1,2,14,15,17]. Perhaps, a more direct role of the antibodies in the increased mortality could be behind the association. First evidences in this direction were reported recently for anti-CCP antibodies in the atheroma plaques [18]. However, some of the findings supporting this mechanism have been contradicted in a subsequent study by our group [45]. Therefore, we still do not have a clear indication of what could be the mechanisms linking the RA autoantibodies with mortality. This applies also to the anti-CarP antibodies. No particular mechanism has been yet explored for them, but as suggested in the single study considering this type of antibodies [9], carbamylated proteins indicate a possible relationship. In effect, carbamylated proteins are increased in patients with chronic kidney disease, in whom they are associated with atherosclerosis and with cardiovascular and all-cause mortality [46,47]. However, we did not know yet whether the levels of protein carbamylation are increased in RA patients or in patients with anti-CarP antibodies, and our results did not show an increase of mortality by circulatory system diseases. An alternative mechanism is suggested by the association of anti-CarP antibodies with diminished lung function measured as FEV1 and with *Pseudomona aeruginosa* infection in patients with cystic fibrosis [48]. This association was attributed to the role of anti-CarP antibodies as marker of neutrophil-driven bronchial inflammation. This interpretation could also pertain to RA because signs of bronchial inflammation are common in seropositive patients with RA [49,50], and even in subjects at risk of RA that have RA autoantibodies [51]. In addition, plasma cells isolated from inducible bronchus-associated lymphoid tissue (iBALT) produce RA autoantibodies [52].

Regarding the possible value of the RA antibodies as biomarkers of increased mortality risk, there are not yet any studies. However, the anti-CarP association with mortality was in the same order of magnitude than smoking in our patients, making it plausible that they could be used in preventive strategies.

Interpretation of our results should reckon some limitations. One that is shared with other studies on this matter is the partial follow-up of the patients. Ideally, all patients should be followed since disease onset to account for all deaths that could be related with RA [3,7]. However, this approach was unfeasible in our case, because both the sample size and the death rate will be much decreased if only new RA patients were included. In addition, confidence in our results is supported by considering that the increase in RA mortality does not take place until several years, about 10 years, after disease onset [7,53]; and because results from established RA patients are similar to the reported for inception cohorts, except for a higher SMR in the established patients [7].

## Conclusions

The anti-CarP antibodies, which are a new type of RA autoantibodies, were associated with increased mortality in Spanish patients with RA, and this association was dominant over the other RA-specific autoantibodies. In addition, it showed specificity for deaths due to respiratory diseases. However, these results require further validation because the relative roles of the different RA autoantibodies are difficult to disentangle due to their high concordance in patients.

## Supporting information

S1 Table Excel table with data from individual patients used in the analysis of survival. (XLS)

## References

[1] McInnes IB, Schett G The pathogenesis of rheumatoid arthritis. N Engl J Med. 2011; 365: 2205–2219. doi: 10.1056/NEJMra1004965 22150039 10.1056/NEJMra1004965

[2] Smolen JS, Aletaha D, McInnes IB Rheumatoid arthritis. Lancet. 2016; 388: 2023–2038. doi: 10.1016/S0140-6736(16)30173-8 27156434 10.1016/S0140-6736(16)30173-8

[3] Wolfe F, Mitchell DM, Sibley JT, Fries JF, Bloch DA, Williams CA, et al The mortality of rheumatoid arthritis. Arthritis Rheum. 1994; 37: 481–494. 8147925 10.1002/art.1780370408

[4] Myasoedova E, Davis JM, Crowson CS, Gabriel SE Epidemiology of rheumatoid arthritis: rheumatoid arthritis and mortality. Curr Rheumatol Rep. 2010; 12: 379–385. doi: 10.1007/s11926-010-0117-y 20645137 10.1007/s11926-010-0117-y

[5] Widdifield J, Bernatsky S, Paterson JM, Tomlinson G, Tu K, Kuriya B, et al Trends in Excess Mortality Among Patients With Rheumatoid Arthritis in Ontario, Canada. Arthritis Care Res (Hoboken). 2015; 67: 1047–1053. 25623141 10.1002/acr.22553

[6] Sparks JA, Chang SC, Liao KP, Lu B, Fine AR, Solomon DH, et al Rheumatoid Arthritis and Mortality Among Women During 36 Years of Prospective Follow-Up: Results From the Nurses' Health Study. Arthritis Care Res (Hoboken). 2016; 68: 753–762. 26473946 10.1002/acr.22752 PMC4944846

[7] Sokka T, Abelson B, Pincus T Mortality in rheumatoid arthritis: 2008 update. Clin Exp Rheumatol. 2008; 26: S35–61. 19026144

[8] Listing J, Kekow J, Manger B, Burmester GR, Pattloch D, Zink A, et al Mortality in rheumatoid arthritis: the impact of disease activity, treatment with glucocorticoids, TNFalpha inhibitors and rituximab. Ann Rheum Dis. 2015; 74: 415–421. doi: 10.1136/annrheumdis-2013-204021 24291654 10.1136/annrheumdis-2013-204021 PMC4316844

[9] Ajeganova S, Humphreys JH, Verheul MK, van Steenbergen HW, van Nies JA, Hafstrom I, et al Anticitrullinated protein antibodies and rheumatoid factor are associated with increased mortality but with different causes of death in patients with rheumatoid arthritis: a longitudinal study in three European cohorts. Ann Rheum Dis. 2016; 75: 1924–1932. doi: 10.1136/annrheumdis-2015-208579 26757747 10.1136/annrheumdis-2015-208579

[10] Humphreys JH, van Nies JA, Chipping J, Marshall T, van der Helm-van Mil AH, Symmons DP, et al Rheumatoid factor and anti-citrullinated protein antibody positivity, but not level, are associated with increased mortality in patients with rheumatoid arthritis: results from two large independent cohorts. Arthritis Res Ther. 2014; 16: 483 doi: 10.1186/s13075-014-0483-3 25471696 10.1186/s13075-014-0483-3 PMC4272533

[11] Kuller LH, Mackey RH, Walitt BT, Deane KD, Holers VM, Robinson WH, et al Determinants of mortality among postmenopausal women in the women's health initiative who report rheumatoid arthritis. Arthritis Rheumatol. 2014; 66: 497–507. doi: 10.1002/art.38268 24574208 10.1002/art.38268 PMC5638120

[12] Mikuls TR, Fay BT, Michaud K, Sayles H, Thiele GM, Caplan L, et al Associations of disease activity and treatments with mortality in men with rheumatoid arthritis: results from the VARA registry. Rheumatology (Oxford). 2011; 50: 101–109. 20659916 10.1093/rheumatology/keq232 PMC3108313

[13] Sihvonen S, Korpela M, Mustila A, Mustonen J The predictive value of rheumatoid factor isotypes, anti-cyclic citrullinated peptide antibodies, and antineutrophil cytoplasmic antibodies for mortality in patients with rheumatoid arthritis. J Rheumatol. 2005; 32: 2089–2094. 16265684

[14] Aletaha D, Bluml S Therapeutic implications of autoantibodies in rheumatoid arthritis. RMD Open. 2016; 2: e000009 doi: 10.1136/rmdopen-2014-000009 27252890 10.1136/rmdopen-2014-000009 PMC4879342

[15] Castelar-Pinheiro Gda R, Xavier RM The Spectrum and Clinical Significance of Autoantibodies in Rheumatoid Arthritis. Front Immunol. 2015; 6: 320 doi: 10.3389/fimmu.2015.00320 26150818 10.3389/fimmu.2015.00320 PMC4471426

[16] Catrina AI, Ytterberg AJ, Reynisdottir G, Malmstrom V, Klareskog L Lungs, joints and immunity against citrullinated proteins in rheumatoid arthritis. Nat Rev Rheumatol. 2014; 10: 645–653. doi: 10.1038/nrrheum.2014.115 25072264 10.1038/nrrheum.2014.115

[17] Humphreys JH, Verheul MK, Barton A, MacGregor AJ, Lunt M, Toes RE, et al Anticarbamylated protein antibodies are associated with long-term disability and increased disease activity in patients with early inflammatory arthritis: results from the Norfolk Arthritis Register. Ann Rheum Dis. 2016; 75: 1139–1144. doi: 10.1136/annrheumdis-2015-207326 26443608 10.1136/annrheumdis-2015-207326 PMC4893092

[18] Sokolove J, Brennan MJ, Sharpe O, Lahey LJ, Kao AH, Krishnan E, et al Brief report: citrullination within the atherosclerotic plaque: a potential target for the anti-citrullinated protein antibody response in rheumatoid arthritis. Arthritis Rheum. 2013; 65: 1719–1724. doi: 10.1002/art.37961 23553485 10.1002/art.37961 PMC3731137

[19] Shi J, Knevel R, Suwannalai P, van der Linden MP, Janssen GM, van Veelen PA, et al Autoantibodies recognizing carbamylated proteins are present in sera of patients with rheumatoid arthritis and predict joint damage. Proc Natl Acad Sci U S A. 2011; 108: 17372–17377. doi: 10.1073/pnas.1114465108 21987802 10.1073/pnas.1114465108 PMC3198314

[20] Shi J, van de Stadt LA, Levarht EW, Huizinga TW, Toes RE, Trouw LA, et al Anti-carbamylated protein antibodies are present in arthralgia patients and predict the development of rheumatoid arthritis. Arthritis Rheum. 2013; 65: 911–915. doi: 10.1002/art.37830 23279976 10.1002/art.37830

[21] Jiang X, Trouw LA, van Wesemael TJ, Shi J, Bengtsson C, Kallberg H, et al Anti-CarP antibodies in two large cohorts of patients with rheumatoid arthritis and their relationship to genetic risk factors, cigarette smoking and other autoantibodies. Ann Rheum Dis. 2014; 73: 1761–1768. doi: 10.1136/annrheumdis-2013-205109 24812286 10.1136/annrheumdis-2013-205109

[22] Shi J, van de Stadt LA, Levarht EW, Huizinga TW, Hamann D, van Schaardenburg D, et al Anti-carbamylated protein (anti-CarP) antibodies precede the onset of rheumatoid arthritis. Ann Rheum Dis. 2014; 73: 780–783. doi: 10.1136/annrheumdis-2013-204154 24336334 10.1136/annrheumdis-2013-204154

[23] Montes A, Regueiro C, Perez-Pampin E, Boveda MD, Gomez-Reino JJ, Gonzalez A Anti-Carbamylated Protein Antibodies as a Reproducible Independent Type of Rheumatoid Arthritis Autoantibodies. PLoS One. 2016; 11: e0161141 doi: 10.1371/journal.pone.0161141 27537849 10.1371/journal.pone.0161141 PMC4990271

[24] Arnett FC, Edworthy SM, Bloch DA, McShane DJ, Fries JF, Cooper NS, et al The American Rheumatism Association 1987 revised criteria for the classification of rheumatoid arthritis. Arthritis Rheum. 1988; 31: 315–324. 3358796 10.1002/art.1780310302

[25] European Commission. Regional Policy. Electronic medical record system IANUS improves regional health care. Accessed on: 2017. http://ec.europa.eu/regional_policy/en/projects/spain/electronic-medical-record-system-ianus-improves-regional-health-care

[26] Spanish National Institute of Statistics (INE). Mortality tables of the Spanish population 1991–2011. Accessed on: 2016. http://www.ine.es/jaxi/Datos.htm?path=/t20/p319a/serie/l0/&file=01003.px

[27] Micallef L, Rodgers P eulerAPE: drawing area-proportional 3-Venn diagrams using ellipses. PLoS One. 2014; 9: e101717 doi: 10.1371/journal.pone.0101717 25032825 10.1371/journal.pone.0101717 PMC4102485

[28] Montes A, Dieguez-Gonzalez R, Perez-Pampin E, Calaza M, Mera-Varela A, Gomez-Reino JJ, et al Particular association of clinical and genetic features with autoimmunity to citrullinated alpha-enolase in rheumatoid arthritis. Arthritis Rheum. 2011; 63: 654–661. doi: 10.1002/art.30186 21360494 10.1002/art.30186

[29] Montes A, Perez-Pampin E, Calaza M, Gomez-Reino JJ, Gonzalez A Association of anti-citrullinated vimentin and anti-citrullinated alpha-enolase antibodies with subsets of rheumatoid arthritis. Arthritis Rheum. 2012; 64: 3102–3110. doi: 10.1002/art.34569 22674012 10.1002/art.34569

[30] Fisher BA, Bang SY, Chowdhury M, Lee HS, Kim JH, Charles P, et al Smoking, the HLA-DRB1 shared epitope and ACPA fine-specificity in Koreans with rheumatoid arthritis: evidence for more than one pathogenic pathway linking smoking to disease. Ann Rheum Dis. 2014; 73: 741–747. doi: 10.1136/annrheumdis-2012-202535 23505239 10.1136/annrheumdis-2012-202535

[31] Hecht C, Englbrecht M, Rech J, Schmidt S, Araujo E, Engelke K, et al Additive effect of anti-citrullinated protein antibodies and rheumatoid factor on bone erosions in patients with RA. Ann Rheum Dis. 2015; 74: 2151–2156. doi: 10.1136/annrheumdis-2014-205428 25115448 10.1136/annrheumdis-2014-205428

[32] van der Woude D, Rantapaa-Dahlqvist S, Ioan-Facsinay A, Onnekink C, Schwarte CM, Verpoort KN, et al Epitope spreading of the anti-citrullinated protein antibody response occurs before disease onset and is associated with the disease course of early arthritis. Ann Rheum Dis. 2010; 69: 1554–1561. doi: 10.1136/ard.2009.124537 20448290 10.1136/ard.2009.124537

[33] Sokolove J, Bromberg R, Deane KD, Lahey LJ, Derber LA, Chandra PE, et al Autoantibody epitope spreading in the pre-clinical phase predicts progression to rheumatoid arthritis. PLoS One. 2012; 7: e35296 doi: 10.1371/journal.pone.0035296 22662108 10.1371/journal.pone.0035296 PMC3360701

[34] England BR, Sayles H, Michaud K, Caplan L, Davis LA, Cannon GW, et al Cause-Specific Mortality in Male US Veterans With Rheumatoid Arthritis. Arthritis Care Res (Hoboken). 2016; 68: 36–45. 26097231 10.1002/acr.22642

[35] Gonzalez A, Icen M, Kremers HM, Crowson CS, Davis JM, Therneau TM, et al Mortality trends in rheumatoid arthritis: the role of rheumatoid factor. J Rheumatol. 2008; 35: 1009–1014. 18412312 PMC2834198

[36] Lopez-Longo FJ, Oliver-Minarro D, de la Torre I, Gonzalez-Diaz de Rabago E, Sanchez-Ramon S, Rodriguez-Mahou M, et al Association between anti-cyclic citrullinated peptide antibodies and ischemic heart disease in patients with rheumatoid arthritis. Arthritis Rheum. 2009; 61: 419–424. doi: 10.1002/art.24390 19333979 10.1002/art.24390

[37] Tomasson G, Aspelund T, Jonsson T, Valdimarsson H, Felson DT, Gudnason V Effect of rheumatoid factor on mortality and coronary heart disease. Ann Rheum Dis. 2010; 69: 1649–1654. doi: 10.1136/ard.2009.110536 19628821 10.1136/ard.2009.110536 PMC3653630

[38] Liang KP, Kremers HM, Crowson CS, Snyder MR, Therneau TM, Roger VL, et al Autoantibodies and the risk of cardiovascular events. J Rheumatol. 2009; 36: 2462–2469. doi: 10.3899/jrheum.090188 19833748 10.3899/jrheum.090188 PMC2837072

[39] Naghavi M, Makela S, Foreman K, O'Brien J, Pourmalek F, Lozano R Algorithms for enhancing public health utility of national causes-of-death data. Popul Health Metr. 2010; 8: 9 doi: 10.1186/1478-7954-8-9 20459720 10.1186/1478-7954-8-9 PMC2873308

[40] Thomas SL, Griffiths C, Smeeth L, Rooney C, Hall AJ Burden of mortality associated with autoimmune diseases among females in the United Kingdom. Am J Public Health. 2010; 100: 2279–2287. doi: 10.2105/AJPH.2009.180273 20864721 10.2105/AJPH.2009.180273 PMC2951969

[41] Walsh SJ, Rau LM Autoimmune diseases: a leading cause of death among young and middle-aged women in the United States. Am J Public Health. 2000; 90: 1463–1466. 10983209 10.2105/ajph.90.9.1463 PMC1447637

[42] Kitas GD, Gabriel SE Cardiovascular disease in rheumatoid arthritis: state of the art and future perspectives. Ann Rheum Dis. 2011; 70: 8–14. doi: 10.1136/ard.2010.142133 21109513 10.1136/ard.2010.142133

[43] Furst DE The risk of infections with biologic therapies for rheumatoid arthritis. Semin Arthritis Rheum. 2010; 39: 327–346. doi: 10.1016/j.semarthrit.2008.10.002 19117595 10.1016/j.semarthrit.2008.10.002

[44] Singh JA, Cameron C, Noorbaloochi S, Cullis T, Tucker M, Christensen R, et al Risk of serious infection in biological treatment of patients with rheumatoid arthritis: a systematic review and meta-analysis. Lancet. 2015; 386: 258–265. doi: 10.1016/S0140-6736(14)61704-9 25975452 10.1016/S0140-6736(14)61704-9 PMC4580232

[45] Montes A, Corrales A, Calaza M, Lopez-Mejias R, Parra JA, Gonzalez-Gay MA, et al Brief report: lack of replication of an association between anti-citrullinated fibrinogen and subclinical atherosclerosis in patients with rheumatoid arthritis. Arthritis Rheumatol. 2015; 67: 2861–2865. doi: 10.1002/art.39302 26246227 10.1002/art.39302

[46] Berg AH, Drechsler C, Wenger J, Buccafusca R, Hod T, Kalim S, et al Carbamylation of serum albumin as a risk factor for mortality in patients with kidney failure. Sci Transl Med. 2013; 5: 175ra129. 10.1126/scitranslmed.3005218 PMC3697767 23467560

[47] Drechsler C, Kalim S, Wenger JB, Suntharalingam P, Hod T, Thadhani RI, et al Protein carbamylation is associated with heart failure and mortality in diabetic patients with end-stage renal disease. Kidney Int. 2015; 87: 1201–1208. doi: 10.1038/ki.2014.429 25671766 10.1038/ki.2014.429 PMC4449819

[48] Skopelja S, Hamilton BJ, Jones JD, Yang ML, Mamula M, Ashare A, et al The role for neutrophil extracellular traps in cystic fibrosis autoimmunity. JCI Insight. 2016; 1: e88912 doi: 10.1172/jci.insight.88912 27777975 10.1172/jci.insight.88912 PMC5070963

[49] Reynisdottir G, Karimi R, Joshua V, Olsen H, Hensvold AH, Harju A, et al Structural changes and antibody enrichment in the lungs are early features of anti-citrullinated protein antibody-positive rheumatoid arthritis. Arthritis Rheumatol. 2014; 66: 31–39. doi: 10.1002/art.38201 24449573 10.1002/art.38201

[50] Reynisdottir G, Olsen H, Joshua V, Engstrom M, Forsslund H, Karimi R, et al Signs of immune activation and local inflammation are present in the bronchial tissue of patients with untreated early rheumatoid arthritis. Ann Rheum Dis. 2016; 75: 1722–1727. doi: 10.1136/annrheumdis-2015-208216 26530319 10.1136/annrheumdis-2015-208216

[51] Demoruelle MK, Weisman MH, Simonian PL, Lynch DA, Sachs PB, Pedraza IF, et al Brief report: airways abnormalities and rheumatoid arthritis-related autoantibodies in subjects without arthritis: early injury or initiating site of autoimmunity? Arthritis Rheum. 2012; 64: 1756–1761. doi: 10.1002/art.34344 22183986 10.1002/art.34344 PMC3319006

[52] Rangel-Moreno J, Hartson L, Navarro C, Gaxiola M, Selman M, Randall TD Inducible bronchus-associated lymphoid tissue (iBALT) in patients with pulmonary complications of rheumatoid arthritis. J Clin Invest. 2006; 116: 3183–3194. doi: 10.1172/JCI28756 17143328 10.1172/JCI28756 PMC1678820

[53] Radovits BJ, Fransen J, Al Shamma S, Eijsbouts AM, van Riel PL, Laan RF Excess mortality emerges after 10 years in an inception cohort of early rheumatoid arthritis. Arthritis Care Res (Hoboken). 2010; 62: 362–370. 20391482 10.1002/acr.20105

